# Distinct effects of blood pressure parameters on Alzheimer’s and vascular markers in 1,952 Asian individuals without dementia

**DOI:** 10.1186/s13195-024-01483-y

**Published:** 2024-06-11

**Authors:** Sungjoo Lee, Si Eun Kim, Hyemin Jang, Jun Pyo Kim, Gyeongmo Sohn, Yu Hyun Park, Hongki Ham, Yuna Gu, Chae Jung Park, Hee Jin Kim, Duk L. Na, Kyunga Kim, Sang Won Seo

**Affiliations:** 1 Department of Digital Health, Samsung Advanced Institute for Health Sciences & Technology (SAIHST), Sungkyunkwan University, 81 Irwon-ro, Gangnam-gu, Seoul, 06351 Republic of Korea; 2 Department of Neurology, Samsung Medical Center, Sungkyunkwan University School of Medicine, 81 Irwon-ro, Gangnam-gu, Seoul, 06351 Republic of Korea; 3 Department of Neurology, Inje University College of Medicine, Haeundae Paik Hospital, Busan, 48108 Republic of Korea; 4 Department of Neurology, Seoul National University Hospital, Seoul National University College of Medicine, Seoul, 03080 Republic of Korea; 5 Neuroscience Center, Samsung Medical Center, Seoul, 06351 Republic of Korea; 6 Alzheimer’s Disease Convergence Research Center, Samsung Medical Center, Seoul, 06351 Republic of Korea; 7 Research Institute, National Cancer Center, Goyang, 10408 Republic of Korea; 8 Biomedical Statistics Center, Research Institute for Future Medicine, Samsung Medical Center, 81 Irwon-ro, Gangnam-gu, Seoul, 06351 Republic of Korea; 9 Department of Data Convergence & Future Medicine, Sungkyunkwan University School of Medicine, 81 Irwon-ro, Gangnam-gu, Seoul, 06351 Republic of Korea; 10 Department of Health Sciences and Technology, SAIHST, Sungkyunkwan University, Seoul, 06351 Republic of Korea; 11 Center for Clinical Epidemiology, Samsung Medical Center, Seoul, 06351 Republic of Korea; 12 Clinical Research Design and Evaluation, SAIHST, Sungkyunkwan University, Seoul, 06351 Republic of Korea

**Keywords:** Blood pressure, Blood pressure variability, Amyloid-beta, Tau, Vascular burden, Hippocampal volume, Cognition

## Abstract

**Background:**

Risk factors for cardiovascular disease, including elevated blood pressure, are known to increase risk of Alzheimer’s disease. There has been increasing awareness of the relationship between long-term blood pressure (BP) patterns and their effects on the brain. We aimed to investigate the association of repeated BP measurements with Alzheimer’s and vascular disease markers.

**Methods:**

We recruited 1,952 participants without dementia between August 2015 and February 2022. During serial clinic visits, we assessed both systolic BP (SBP) and diastolic BP (DBP), and visit-to-visit BP variability (BPV) was quantified from repeated measurements. In order to investigate the relationship of mean SBP (or DBP) with Alzheimer’s and vascular markers and cognition, we performed multiple linear and logistic regression analyses after controlling for potential confounders (Model 1). Next, we investigated the relationship of with variation of SBP (or DBP) with the aforementioned variables by adding it into Model 1 (Model 2). In addition, mediation analyses were conducted to determine mediation effects of Alzheimer’s and vascular makers on the relationship between BP parameters and cognitive impairment.

**Results:**

High Aβ uptake was associated with greater mean SBP (β = 1.049, 95% confidence interval 1.016–1.083). High vascular burden was positively associated with mean SBP (odds ratio = 1.293, 95% CI 1.015–1.647) and mean DBP (1.390, 1.098–1.757). High tau uptake was related to greater systolic BPV (0.094, 0.001–0.187) and diastolic BPV (0.096, 0.007–0.184). High Aβ uptake partially mediated the relationship between mean SBP and the Mini-Mental State Examination (MMSE) scores. Hippocampal atrophy mediated the relationship between diastolic BPV and MMSE scores.

**Conclusions:**

Each BP parameter affects Alzheimer’s and vascular disease markers differently, which in turn leads to cognitive impairment. Therefore, it is necessary to appropriately control specific BP parameters to prevent the development of dementia. Furthermore, a better understanding of pathways from specific BP parameters to cognitive impairments might enable us to select the managements targeting the specific BP parameters to prevent dementia effectively.

**Supplementary Information:**

The online version contains supplementary material available at 10.1186/s13195-024-01483-y.

## Background

Alzheimer’s disease (AD) is characterized by amyloid-beta (Aβ) and tau deposition [1]. Neuronal injury, neuroinflammation and vascular disease also play a crucial role in the pathogenesis of AD [2–4]. Cerebral small vessel disease (CSVD) is characterized by extensive white matter hyperintensities (WMH) [5]. Pathological studies have demonstrated that dementia with AD-type is more frequently associated with concurrent CSVD loads compared to other neurodegenerative illnesses [5]. The advancement of Aβ and tau positron emission tomography (PET) also enabled us to detect AD imaging markers in patients with CSVD lesions [4]. In fact, AD combined with CSVD is reported to be the most prevalent mixed pathology [6–8]. Out of the total number of individuals with dementia, 38.0% (19 out of 50) have both AD and infarcts, 30.0% (15 out of 50) have only pure AD, and 12% (6 out of 50) have vascular dementia [6].

Epidemiological studies have shown that hypertension is associated with increased risk for dementia [4, 9–11]. Increased mean blood pressure (BP) promotes white matter alterations, resulting in the development of WMH [12]. It is also associated with an increased rate of brain atrophy with or without the mediation of WMH [13, 14]. Furthermore, several studies suggest that the presence of hypertension may enhance the deposition of Aβ and tau in the brain [15, 16]. More recently, BP variability (BPV) has been related to an increased risk of dementia [17], which highlights the importance of understanding the role of various BP parameters in the development of dementia. Notably, there has been a growing focus on BPV over months to years (e.g., visit-to-visit BPV), as a modifiable risk factor for cerebrovascular illness and cognitive decline, independent of average BP levels [18–21]. However, the associations between specific BP parameters and markers of AD and vascular disease have not been extensively established yet. Specific BP parameters might have various associations with markers of AD and vascular disease, eventually contributing to the development of dementia [22, 23]. In addition, recent study has shown that managing patients at increased risk for cardiovascular events, intensive treatment to reduce BP was linked to decreased rates of fatal and nonfatal cardiovascular events, as well as overall mortality, compared to standard treatment [24, 25]. Thus, in the treatment of hypertension, it is necessary to target specific BP parameters to prevent the development of dementia. Furthermore, a better understanding of pathways from specific BP parameters to cognitive impairment might enable us to select the specific medications targeting the specific BP parameters to prevent dementia effectively.

Thus, in the present study, we investigated the effects of specific BP parameters on AD and vascular disease markers in individuals without dementia. Furthermore, we determined whether these AD and vascular disease markers might mediate the relationship between specific BP parameters and cognitive impairment. We hypothesized that each BP parameter would affect Aβ, tau uptake, hippocampal atrophy and development of WMH differently, which in turn leads to cognitive impairment.

## Methods

### Study participants

**Fig. 1:**
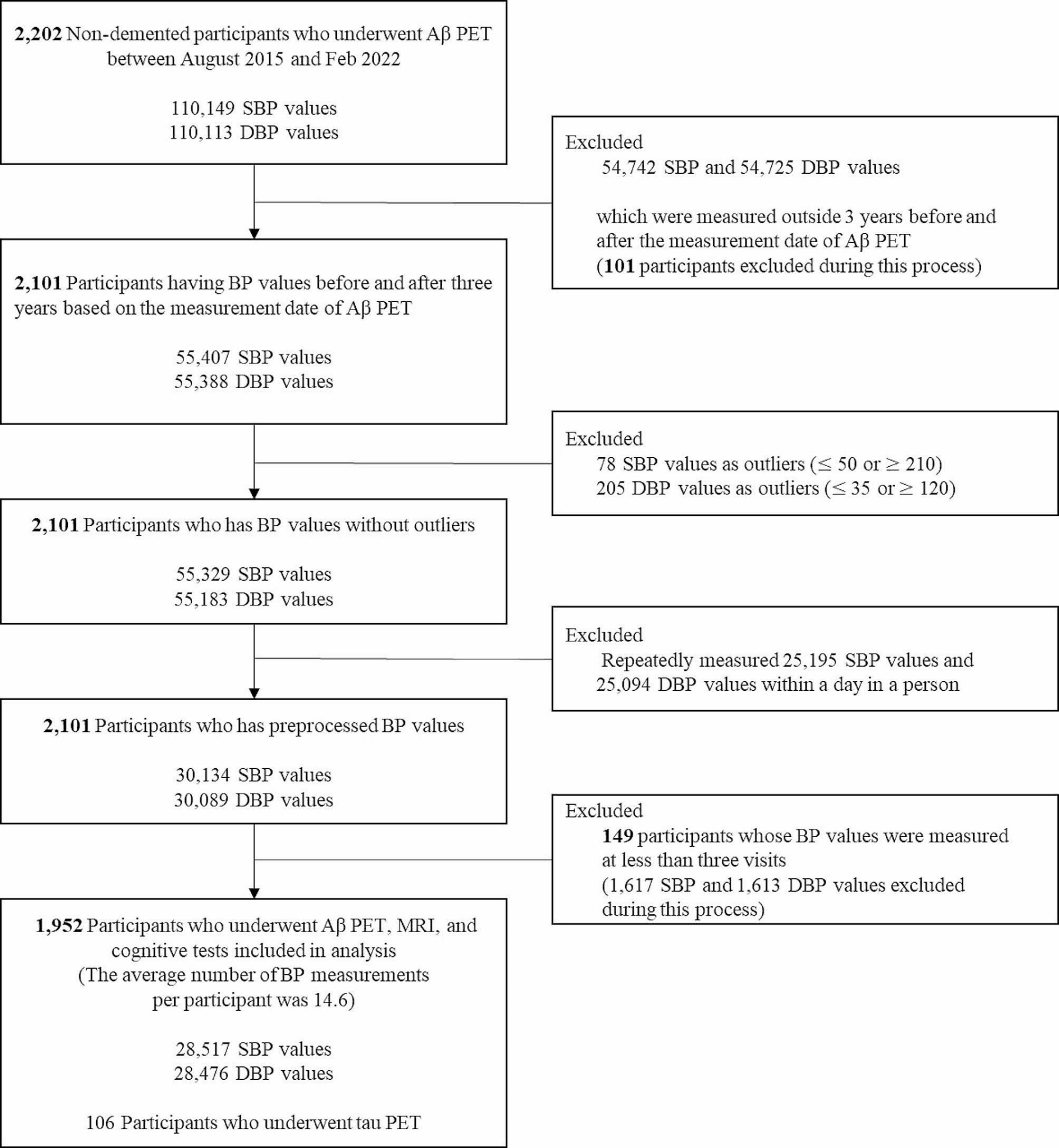
Study flow chart Abbreviations: Aβ, amyloid-beta; PET, positron emission tomography; SBP, systolic blood pressure; DBP, diastolic blood pressure; BP, blood pressure

We enrolled 2,202 individuals without dementia who attended the memory clinic at Samsung Medical Center (SMC) in South Korea from August 2015 to February 2022 (Fig. 1). Health professionals conducted medical assessments utilizing standardized protocols. Every subject got a comprehensive evaluation that included a neurological examination, cognitive testing, standard blood tests, brain magnetic resonance imaging (MRI), and Aβ [18 F-florbetaben (FBB) or 18 F-flutemetamol (FMM)] PET scans. During the tests, we identified the vascular risk factors, including hypertension (defined as a self-reported medical history of hypertension or currently taking antihypertensive drugs), diabetes mellitus (defined as a self-reported history of diabetes mellitus or currently taking insulin or oral antidiabetic medications). The blood tests conducted on all individuals encompassed a complete blood count, blood chemistry analysis, vitamin B12/folate levels, syphilis serology, thyroid function panel, and apolipoprotein E (APOE) genotyping. Among them, 106 participants underwent tau [18 F-flortaucipir (FTP)] PET scans. We excluded participants with structural lesions such as brain tumor, large territorial infarct, and intracranial hemorrhage, as well as those with other causes of neurodegenerative disease including Parkinson’s disease, Lewy body dementia, progressive supranuclear palsy, cortico-basal syndrome, and frontotemporal dementia. Participants were further classified into cognitively unimpaired (CU) and mild cognitive impairment (MCI) groups. CU individuals in the study met the following criteria: (1) they had no medical history that would likely impact their cognitive function, as determined by Christensen’s health screening criteria [26], (2) they did not show any objective cognitive impairment in any cognitive domain, as measured by a comprehensive neuropsychological test battery, with scores at least − 1.0 standard deviation (SD) above age-adjusted norms, and (3) they were able to independently perform activities of daily living [27]. MCI participants met the following criteria [28]: (1) they or their caregiver reported subjective cognitive complaints; (2) they exhibited objective memory impairment below − 1·0 SD on verbal or visual memory tests; (3) they did not have significant impairment in their ability to do daily activities; and (4) they did not have dementia. When distinguishing between MCI and dementia, we used the Seoul-Instrumental Activities of Daily Living, and the cut-off score was 8 [29, 30].

This study was approved by the Institutional Review Board of SMC (IRB No: 2019-11-177). Participants and their caregivers provided written informed consent for participating in the study and publication.

### Cognitive assessment

The participants completed a standardized neuropsychological test battery known as the Seoul Neuropsychological Screening Battery (SNSB). This battery included tests that assessed attention, language, visuospatial abilities, memory, and frontal/executive functions [31]. The scored tests included the Digit Span Forward (DSF), Korean version of the Boston Naming Test (K-BNT), Rey-Osterrieth Complex Figure Test (RCFT: copying, immediate and 20-minute delayed recall, and recognition), Seoul Verbal Learning Test (SVLT: immediate, 20-minute delayed recall and recognition), phonemic and semantic Controlled Oral Word Association Test (COWAT), and the Stroop Test (color reading). The DSF was utilized to evaluate the level of attentiveness. Verbal and nonverbal learning and memory were evaluated using SVLT and RCFT. The K-BNT and RCFT assessments were conducted to assess language and visuospatial function, respectively. The phonemic and semantic COWAT and the Stroop Test were conducted to assess frontal/executive function. In addition, all participants underwent the Mini-Mental State Examination (MMSE) in order to evaluate their overall cognitive performance [32]. Abnormal scores were defined as those that fell below − 1.0 standard deviations from the age- and education-adjusted norms. The tests were conducted by experienced staff and overseen by clinical neuropsychologists who are certified by the board.

### BP parameters

For each participant, an observation period of BP was defined as a period within three years before and after inspecting Aβ PET. We extracted systolic BP (SBP) and diastolic BP (DBP) records of all participants during their observation periods from the Clinical Data Warehouse of SMC. According to the guidelines in which the hospital follows [33, 34], the BP measurement was conducted with appropriate preparation, which included resting for 5 min in a quiet room and abstaining from smoking, alcohol, and caffeine for 30 min before to the measurement. Additionally, the cuff was placed at the level of the heart to ensure optimal posture. During each clinic visit, the patient’s seated BP and pulse were measured using an automated device. If needed, manual devices were used. The measurements were taken at regular intervals of 1–2 min [33]. We excluded patients with very severe hypertension or hypotension [24, 34–36]. Therefore, outliers of SBP (≤ 50 or ≥ 210) and DBP (≤ 35 or ≥ 120) values were removed. There were 1.84 SBP (range = 1–38) and 1.83 DBP (range = 1–36) records per participant on average in a single visit. When a participant had multiple records in a single day, the median of those records was selected as a BP value for the day. With this process, all subjects became have only one BP record per visit but could have multiple BPs over the entire follow-up time. For the next step, we excluded participants who had less than three BP values because BPV was not computable. Finally, 1,952 participants (729 CU and 1,223 MCI) were included in the analysis (Fig. 1). The average number of visits per participant during the entire follow-up time was 14.6 (range = 3-115).

Two different BP parameters, visit-to-visit mean BP and BPV for each subject, were considered in this study. Mean BPs and BPVs were defined as the averages and SDs of all BP values within a subject, respectively. SD was selected as the measure for BPV since the SD was a common measure for visit-to-visit variability [37].

First, the within-participant mean SBP and mean DBP were assessed with the averages of SBP and DBP values, respectively. Second, the within-participant systolic and diastolic BPVs were assessed with the SDs of SBP and DBP values since the SD is a common measure for visit-to-visit variability.

### Brain MRI acquisition and measurement of hippocampal volumes

All participants received three-dimensional (3D) T1 turbo field echo images and 3D fluid-attenuated inversion recovery (FLAIR) at SMC utilizing a 3.0T MRI scanner (Philips 3.0T Achieva; Philips Healthcare, Andover, MA, USA), as previously described [38].

To measure hippocampal volumes (HV), we employed an automated method that involved a graph cut algorithm paired with an atlas-based segmentation and morphological opening as described in an previous study [39]. Intracranial volume (ICV) was determined by quantifying the combined the volumes of voxels contained within the the brain mask after the removal of the skull.

### Assessment of vascular burden

The WMH visual rating scale, developed by the Clinical Research Center for Dementia of South Korea, was utilized to examine the presence of WMH in the deep subcortical and periventricular areas on FLAIR images [38–40]. We defined vascular positivity (V+) as severe levels of WMH visual rating scales according to our classification system for ischemia [39]. This classification system differentiates the intensity of CSVD markers, such as the volume of WMH [39]. In summary, deep WMH were categorized as D1 (< 10 mm), D2 (10–25 mm), or D3 (≥ 25 mm) according to the lesions’ longest diameter. The classification of periventricular WMH was based on their maximum length measured perpendicular to the ventricle (cap) and horizontally (band). WMH were categorized as P1 if their length was less than 5 mm, P2 if it ranged from 5 to 10 mm, and P3 if it was equal to or greater than 10 mm. There was a total of 9 cells resulting from the combination of D and P ratings. The overall severity of WMH (minimal, moderate, and severe) was determined based on the following combinations of D and P ratings: minimum (D1P1, D1P2), moderate (D1P3, D2P1, D2P2, D2P3, D3P1, D3P2), and severe (D3P3) [40]. In order to evaluate the interrater reliability of our WMH visual rating, we randomly assigned 100 FLAIR images and had 3 experienced neurologists perform a visual rating of the WMH severity. The interrater agreement for the overall severity of WMH was excellent, with a Fleiss k value of 0.84.

### Amyloid PET imaging acquisition and analysis

Each participant had either FBB or FMM PET at SMC using a Discovery STe PET/CT scanner (GE Medical Systems, Milwaukee, WI, USA) in 3D scanning mode. This mode examines 47 slices of 3.3 mm-thickness that cover the entire brain [41]. CT images were obtained using a 16-slice helical CT scanner with a 140 keV energy level and 80 mA current. This section width of each image was 3.75 mm, and these images were used for attenuation correction. As per the guidelines provided by the makers of the ligands, a 20-min emission PET scan was conducted using dynamic mode (consisting of 4 × 5 min frames). This scan was performed 90 min after injecting an average dose of 311.5 MBq of FBB or 185 MBq of FMM. The 3D PET images were created using the ordered-subsets expectation-maximization (OSEM) algorithm (FBB iterations = 4 and subset = 20; FMM iterations = 4 and subset = 20). The images were reconstructed in a 128 × 128 × 48 matrix with a voxel size of.

2.00 × 2.00 × 3.27 mm3. The PET data were aligned with individual 3D-T1 weighted MR images, which were then standardized to the T1-weighted MNI-152 template utilizing Statistical Parametric Mapping (SPM) 8.

In our previous study, to improve the prediction of prognosis and early detection, we developed an MRI-based regional modified Centiloid (rdcCL) method that harmonizes the overall and regional Aβ uptake among Aβ ligands [42]. The reference region used in the Centiloid pipeline was the whole cerebellum. More details of the analysis method followed are in the original Centiloid project paper and a previous paper [42, 43]. MRI and PET images underwent spatial normalization using the transformation parameters obtained from the SPM8 [44].

### Tau PET imaging acquisition and analysis

^18^F-Flortaucipir PET images were obtained using a Discovery STE PET/CT (GE Healthcare) at SMC and a Biographic mCT PET/CT scanner (Siemens Medical Solutions) at Gangnam Severance Hospital. Following the injection of intravenous bolus doses of 280 MBq ^18^F-flortaucipir, PET images were obtained during a 20-minute acquisition period at 80 min post-injection. Prior to the PET scan, we affixed a head holder to reduce head movement and obtained brain CT images for the purpose of attenuation correction. PET images were reconstructed in a three-dimensional matrix with dimensions of 128 × 128 × 47 with 2.00 × 2.00 × 3.27 mm voxel size at SMC and in a 256 × 256 × 223 matrix with 1.591 × 1.591 × 1 mm voxel size at Gangnam Severance Hospital using the OSEM algorithm (iteration = 6 and subset = 16).

Flortaucipir PET images were realigned and co-registered to the structural MRIs of participants using SPM12. To perform regional standardized uptake value ratio (SUVR) analysis, FreeSurfer 6.0 (http://surfer.nmr.mgh.harvard.edu/) was used to generate region of interest (ROI) masks in the native spaces. Cerebellar gray matter was used as the reference region. For partial volume correction (PVC) of ROI data on the flortaucipir PET images, we used the region-based voxel-wise correction (RBV) method according to the PETPVC toolbox [45]. Consequently, we computed the regional SUVR with the PVC in 41 cortical areas. Then, we created bilateral Braak stage ROIs that anatomically represented the Braak staging regions associated with tau pathology in AD [46–50]. By combining non-overlapping ROIs from FeeSurfer, we established Braak ROIs categorized as Braak I/II, Braak III/IV, and Braak V/VI [51]. Specifically, flortaucipir SUVR using PETPVC applied Braak III/IV ROI [parahippocampal, fusiform, lingual, amygdala, inferior and middle temporal, temporal pole, thalamus, caudal anterior and rostral anterior cingulate, isthmus cingulate, posterior cingulate, and insula] was used.

### Statistical analysis

The baseline characteristics were summarized using the mean ± SD for continuous variables and frequency (percentage) for categorical variables. First, to investigate the relationships of each mean BP with AD markers such as Aβ uptake, tau uptake, and HV, or cognition scores from the MMSE, we used multivariable linear regression models with an adjustment for potential confounders (Model 1). However, multivariable logistic regression analyses were conducted to identify the association between mean BP and WMH because its type was binary. Variables having *P*-values ≤ 0.2 in univariable analyses were selected as potential confounders. While age, sex, duration of education, hypertension, diabetes mellitus, and ICV were selected for HV, the presence of the APOE4 allele was selected instead of ICV for other markers. Second, in Model 2, SDs of BP were included as independent variables to identify the relationships of BPV with AD, CSVD markers and cognitive scores respectively, further adjusting for mean SBP and mean DBP. Third, we added interaction term of BP parameters and the presence of hypertension in the multivariable regression models. It was performed to determine whether the presence of hypertension has moderated effect on the association of BP parameters with AD markers, CSVD marker, or MMSE scores.

All results were presented as the regression coefficients (βs) and odds ratios (ORs) with 95% confidence intervals (CIs) from multivariable linear and logistic regression analyses, respectively. Log transformation was used to revise the skewed distribution of Aβ uptake before analyses, and all results were reported in the original scale. Especially, the results of multivariable linear regression analyses were reported with risk ratios (RRs) that are defined as the inverse-log transformed values of regression coefficients.

Causal mediation analyses were performed to examine the mediation effects of AD and vascular disease markers on the relationships between BP parameters and MMSE scores. Among BP parameters, candidate exposures were selected as those showing potential association with MMSE scores (*P*-values < 0.1) in the multivariable linear regression analyses with adjustment (Model 1 or 2). Among AD and vascular disease markers, candidate mediators were chosen as those having potential association with selected exposure (*P*-values < 0.1) in the multivariable analyses with adjustment (Model 1 or 2). We estimated the natural direct (NDE) and indirect effects (NIE) of BP parameters on MMSE scores using the imputation strategies of Vansteelandt based on the counterfactual framework with the medflex (version 0.6-7) package in R software [52]. Bootstrap-based standard errors were applied to calculate 95% CIs and *P*-values of NDEs and NIEs. The total effect was defined as the summation of NIE and NDE. By dividing NIE by the total effect, we assessed the proportion mediated that indicates the portion of the indirect effect among the total effect of the BP parameters on MMSE scores. Freedman’s proportion explained was also calculated to identify the extent of surrogacy of AD and vascular disease markers when only NIE was significant [53]. Statistical significance was declared with a two-sided *p*-value < 0.05. All analyses were performed using R 4.1.0 (Vienna, Austria; http://www.R-project.org).

## Results

### Clinical characteristics of participants

The demographic and clinical characteristics of the participants are shown in Table 1. Among 1,952 participants, 729 (37.4%) were CU and 1,223 (62.6%) were MCI. The mean age of participants was 71.4 ± 8.7 years and 1126 (57.7%) were females. In relation to vascular risk factors, the subjects had a prevalence of 955 (49.5%) for hypertension and 409 (21.2%) for diabetes mellitus, respectively.

**Table 1:** Baseline characteristics of study participants

Variables	Total (*N* = 1,952)
***Demographic and clinical characteristics***	
Age (years)	71.4 ± 8.7
Female	1,126 (57.7)
Duration of education (years)	12.1 ± 4.7
Diagnosis	
CU	729 (37.4%)
MCI	1,223 (62.6%)
Ligand type	
^18^F-Florbetaben	566 (29.0%)
^18^F-Flutemetamol	1386 (71.0%)
Global Centiloid	42.6 ± 52.0
APOE ε4 carrier (missing *N* = 37)	677 (35.4%)
Diagnosis of hypertension (missing *N* = 22)	955 (49.5%)
Diagnosis of diabetes mellitus (missing *N* = 22)	409 (21.2%)
ICV (cm^3^)	1,320 ± 130
***BP measurements per participant***	
Observation period (months)	37.2 ± 18.7
The average of intervals between BP values (months)	3.2 ± 1.8
Number of BP values	14.6 ± 12.6
SBP parameters (mm Hg)	
Mean SBP	130.0 ± 12.0
SD of SBP	12.2 ± 4.2
DBP parameters (mm Hg)	
Mean SBP	69.6 ± 8.0
SD of SBP	7.8 ± 2.7

Continuous or categorical variables were represented as mean ± standard deviation or frequency (%), respectively

Abbreviations: N, number of participants; CU, cognitively unimpaired; MCI, mild cognitive impairment; APOE ε4, apolipoprotein E ε4 allele; ICV, Intracranial volume; BP, blood pressure; SBP, systolic blood pressure; DBP, diastolic blood pressure; SD, standard deviation

### Association of BP parameters with Alzheimer’s and vascular disease markers

**Fig. 2:**
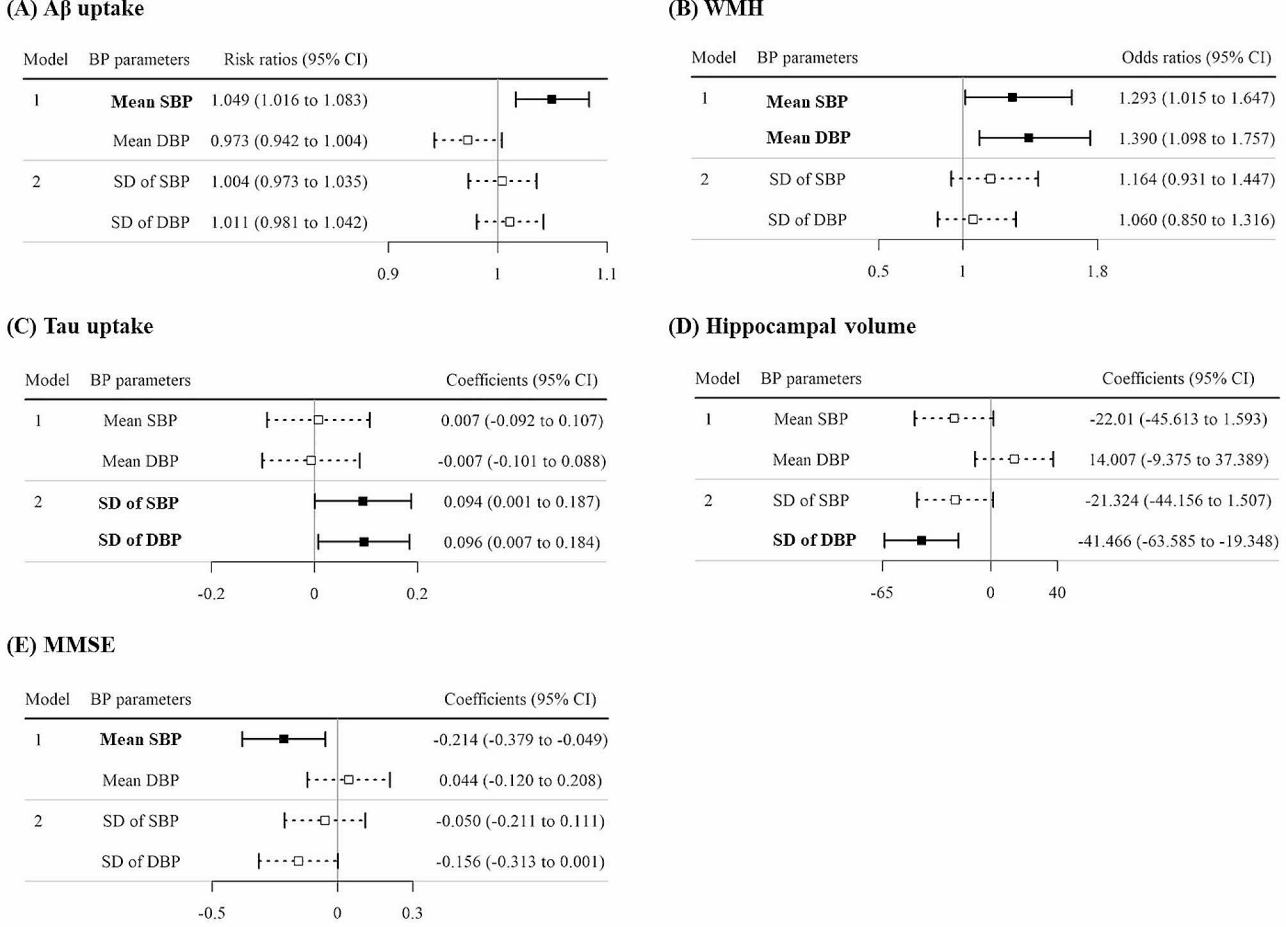
Forest plots showing the association of BP parameters with Aβ uptake, WMH, tau uptake, hippocampal volume and MMSE expressed as risk ratios, odds ratios, or regression coefficients with the 95% confidence intervals. Bold lines in forest plots represent statistically significant associations Abbreviations: Aβ, amyloid-beta; BP, blood pressure; SBP, systolic blood pressure; DBP, diastolic blood pressure; SD, standard deviation; CI, confidence interval; Coefficients, regression coefficients; WMH, white matter hyperintensities; MMSE, Mini-Mental State Examination

The forest plot in Fig. 2A shows the risk of Aβ uptake by BP parameters. The global Aβ Centiloid was positively associated with mean SBP (RR = 1.049, 95% CI, 1.016 to 1.083, *P* = 0.003). There was no association between the global Aβ Centiloid with the SDs of SBP (*P* = 0.805) and DBP (*P* = 0.476) or mean DBP (*P* = 0.085) (Supplementary Table 1).

In Fig. 2B, the forest plot shows the risk of developing WMH by BP parameters using the ORs. The risk elevation in WMH was associated with high mean SBP (OR = 1.293, 95% CI 1.015 to 1.647, *P* = 0.038) and mean DBP (OR = 1.390, 95% CI 1.098 to 1.757, *P* = 0.006). No association of WMH was found with SDs of SBP (*P* = 0.181) and DBP (*P* = 0.600) (Supplementary Table 2).

Figure 2C shows the expected tau uptake by BP parameters. Whereas the tau uptake did not show associations with mean BPs (*P* = 0.882 for SBP; *P* = 0.888 for DBP), it had positive associations with SDs of SBP (β = 0.094, 95% CI 0.001 to 0.187, *P* = 0.049) and DBP (β = 0.096, 95% CI 0.007 to 0.184, *P* = 0.034) (Supplementary Table 3).

The risk of hippocampal atrophy by BP parameters is shown in Fig. 2D. While other BP parameters were not associated (*P* = 0.068 for mean SBP; *P* = 0.240 for mean DBP; *P* = 0.067 for SD of SBP), a low HV was associated with high SD of DBP (β = -41.466, 95% CI -63.585 to -19.348, *P* = 0.0002) (Supplementary Table 4).

As a result of multivariable regression model of MMSE scores in Fig. 2E, mean SBP showed the association with MMSE (β = -0.214, 95% CI -0.379 to -0.049, *P* = 0.011). SD of DBP tended to be associated with MMSE (β = -0.156, 95% CI -0.313 to 0.001 *P* = 0.051). (Supplementary Table 5).

There were no interactive effects of history of hypertension and BP parameters on AD and CSVD markers and MMSE scores (Supplementary Tables 6–10).

### Association of BP parameters with MMSE scores through the mediation of Alzheimer’s and vascular disease markers

**Fig. 3:**
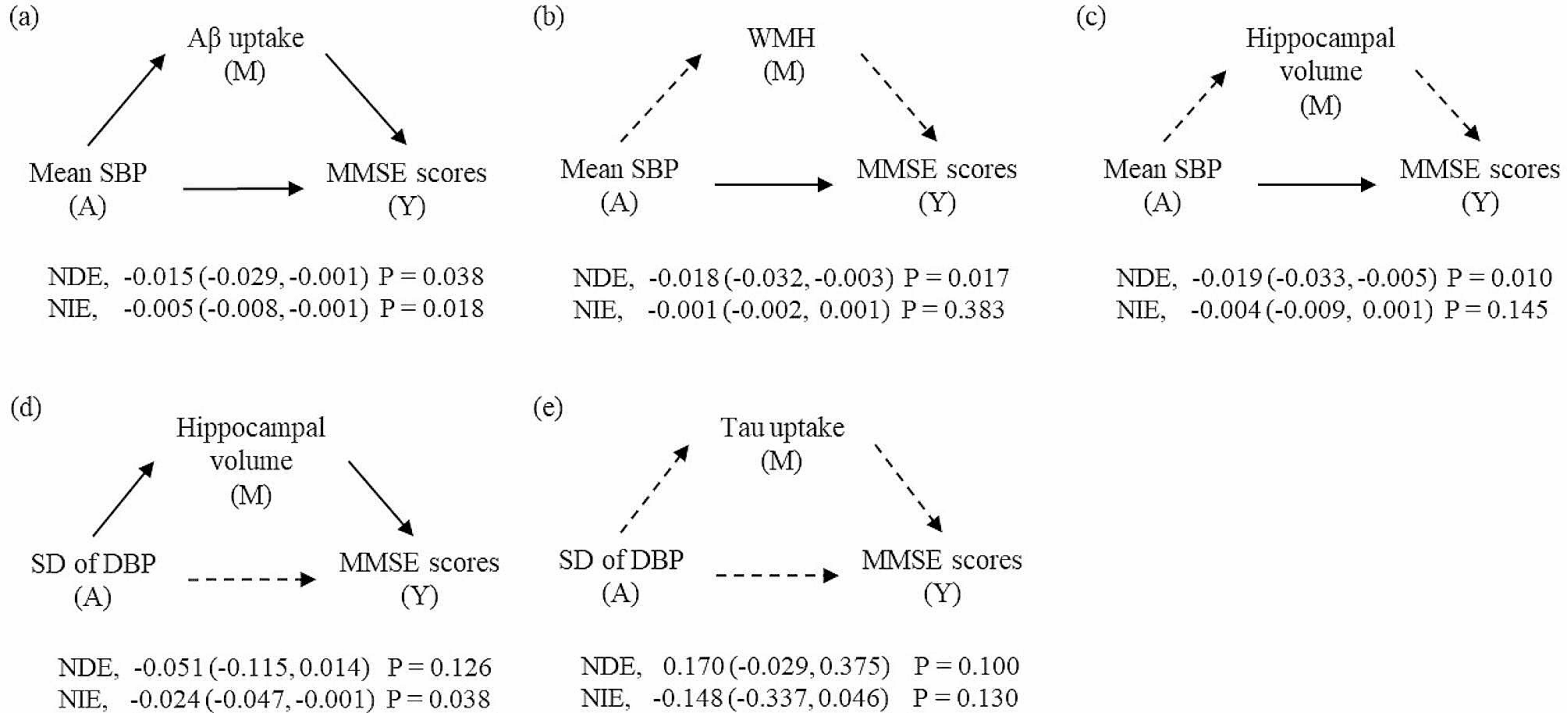
Causal relationship diagrams showing the natural direct and indirect effects of BP parameters on MMSE scores with mediation by Alzheimer’s and vascular markers. A, M, and Y in the diagram indicate the exposure, mediator, and outcome variables, respectively. Natural direct and indirect effects are represented as estimates (95% confidence intervals) with *P*-values. The solid or dashed lines indicate whether the effects are statistically significant or not Abbreviations: Aβ, amyloid-beta; WMH, white matter hyperintensities; SBP, systolic blood pressure; DBP, diastolic blood pressure; SD, standard deviation; MMSE, Mini-Mental State Examination; NDE, natural direct effect; NIE, natural indirect effect

The relationship between mean SBP and MMSE scores was partially mediated by Aβ deposition (proportion mediated = 23.8%, 95% CI 22.7–44.2%), and was explained both directly and indirectly (NDE = -0.015, 95% CI -0.029 to -0.001, *P* = 0.038; NIE = -0.005, 95% CI -0.008 to -0.001, *P* = 0.018) (Fig. 3A). The direct effect between mean SBP and MMSE was observed (NDE = -0.018, 95% CI -0.032 to -0.003, *P* = 0.017), but there was no mediated effect of WMH (Fig. 3B). Likewise, only direct effect was identified between mean SBP and MMSE scores when the mediator was selected as HV (NDE = -0.019, 95% CI -0.033 to -0.005, *P* = 0.010; Fig. 3C). The relationship between SD of DBP and MMSE scores was mediated by HV (NIE = -0.024, 95% CI -0.047 to -0.001, *P* = 0.038) (Fig. 3D), and HV showed partial surrogacy (proportion explained = 0.32). Any significant effect was not observed when the exposure, mediator, and outcome were SD of DBP, tau uptake, and MMSE scores, respectively (Fig. 3E**).**

## Discussion

In the present study, we investigated the effects of specific BP parameters on AD and vascular disease markers in carefully phenotyped and large-sized cohorts who underwent molecular and structural imaging. Our major findings are as follows. First, high Aβ uptake was associated with high mean SBP. Second, high vascular burden was associated with high mean SBP and DBP. Third, high tau uptake was associated with higher SBP and DBP variability. Fourth, higher DBP variability was predictive of hippocampal atrophy. Finally, high Aβ uptake partially mediated the relationships between high mean SBP and cognitive impairment. Hippocampal atrophy mediated the relationships between higher DBP variability and cognitive impairment. Taken together, our findings suggest that among the four BP parameters, each BP parameter affects AD and vascular disease markers differently, which in turn leads to cognitive impairment. Furthermore, our findings highlight the importance of targeting modifiable BP parameters to prevent the development of dementia effectively.

Our first major finding was that high Aβ uptake was associated with high mean SBP. Our finding is in line with previous studies suggesting that SBP increased Aβ burden in non-ε4 carriers [15]. Identifying the precise mechanism by which high SBP may contribute to the development of AD is challenging. However, our findings might be related to the fact that increased SBP is associated with microvascular damage and a compromised blood-brain barrier (BBB) [54]. The BBB plays a crucial function in the elimination of Aβ from the brain [55], therefore the impairment of the BBB could potentially lead to increased Aβ accumulation in the brain [56, 57].

We found that high vascular burden was associated with high mean SBP and mean DBP. According to a meta-analysis, SBP and DBP have a strong and largely consistent association with the severity of WMH [58]. It has been postulated that increased SBP and DBP might develop arterial stiffness, which in turn leads to alterations in cerebrovascular autoregulation, eventually resulting in decreased cerebral blood flow [59].

In the present study, higher SBP and DBP variabilities tend to be associated with high tau uptake. Our finding is consistent with a recent study showing that higher SBP and DBP variabilities were related to increased tau uptake in the temporal region [60]. Our finding might be explained by several hypotheses including ischemia-induced activation of cyclin-dependent kinase 5 (CDK-5) and glycogen synthase kinase 3β (GSK-3β), eventually resulting in the hyperphosphorylation of tau [61, 62].

Another notable finding is that a higher DBP variability was predictive of greater hippocampal atrophy. The results of our research align with a prior study that shown a correlation between a higher DBP variability over three years and a more pronounced decrease in brain atrophy [63]. This might be explained by several hypotheses including hemodynamic instability, inflammation and oxidative stress, arterial stiffness, small vessel disease, and autonomic dysfunction. Specifically, elevated BPV leads to fluctuations in cerebral blood flow, which in turn produce episodes of excessive and insufficient blood supply to the brain. This results in harm to the endothelial cells and smooth muscles in the brain, which in turn triggers damage to the neurovascular unit and initiates neuronal injury [22, 64]. The results further support the concept that BPV has a significant impact on brain structures. This is consistent with a previous study that proposed individuals with higher BPV undergo a faster neuronal shrinkage than expected during the normal aging process [65].

The final major finding was that specific BP parameters affect cognitive impairment through specific pathways. That is, high mean SBP affected cognitive impairment with and without the mediation of high Aβ uptake. Higher DBP variability also affected cognitive impairment with the mediation of hippocampal atrophy. Therefore, our findings suggested that to prevent the progression to dementia, the clinicians should consider specific BP parameters, including mean SBP levels and DBP variability, for the treatment of BP. In addition, imaging markers related to specific BP parameters could also be taken into consideration when monitoring the effects of BP treatment. We also did not find there were interactive effects of history of hypertension and BP parameters on AD and CSVD markers. It suggested that the effect of each BP parameter on these markers would not depend on the presence of hypertension. Thus, it is necessary to appropriately control specific BP parameters to prevent the development of dementia regardless of the history of hypertension.

The strengths of the present study include its prospective setting and the use of standardized Aβ and tau PET and MRI protocols in carefully phenotyped cohorts with a large number of participants who do not have dementia. However, there are several limitations in this study. First, we used Aβ and tau PET uptakes, along with the presence of severe WMH and cortical thickness on MRI, to assess Aβ, tau, CSVD, and neurodegenerative pathologies due to the unavailability of pathological confirmation. Thus, it is not possible to take into account other neurodegenerative disorders that contribute to neurodegeneration, such as tau, transactive response DNA-binding protein (TDP-43), argyrophilic grain disease, and hippocampal sclerosis. Second, due to the inherent difficulties associated with conducting a retrospective cohort study, information regarding the neuroimaging biomarker status of individuals at the beginning of the study was not accessible. Consequently, we were unable to determine their causal relationships. Nevertheless, a retrospective cohort research may be a realistic alternative due to the gradual emergence of changes in neuroimaging biomarkers and the cost associated with their evaluation. Third, due to the retrospective acquisition of BP parameters from the clinical data warehouse, there were variations in the length of follow-up among participants, despite controlling for the duration of follow-up in the process of calculating BPV changes. However, these findings may accurately represent the practical circumstances in real-life environments, thus providing valuable real-world evidence for healthcare decision-making. Next, throughout the period of the MRI scanning, we were unable to account for changes in medication or subclasses of antihypertensive medications. Because different antihypertensive treatments have distinct methods of lowering BP or components of BP, they may also have distinctive impacts on brain [66]. In addition, the range of BPV in the current sample could be limited as participants with a SBP (≤ 50 or ≥ 210) and DBP (≤ 35 or ≥ 120) were excluded. Therefore, it could be difficult to generalize our findings with solely based on this study. Moreover, we did not find the mediation effects of vascular burden on the relationships between BP parameters and cognitive impairment. Previously, our WMH visual rating scale represented cerebral small vessel diseases such as such as the volume of WMH [39]. However, our WMH scale did not seem to fully represent microvascular damage, compromised BBB and alterations in autoregulation. Vascular burden on the association between BP and cognitive impairment could be better explained by applying quantification of WMH volume in the future study. Another limitation is that the number of participants with tau PET was small compared to those with Aβ PET. This might be related to our inability to demonstrate mediating effects of tau on the association between SD of DBP and HV. Finally, we used assumptions regarding confounders in causal mediation analysis. While it is not feasible to completely eliminate all unmeasured factors that could influence the results, we made an effort to incorporate all conceivable variables that could have a substantial impact on the aforementioned associations.

In conclusion, each BP parameter differently affects AD and vascular disease markers, which in turn leads to cognitive impairment. Furthermore, our findings highlight the importance of targeting modifiable BP parameters to prevent the development of dementia. In future studies, it is necessary to continue collecting long-term repeated measurement data on BP, cognitive, structural, and functional brain changes to develop a strong evidence-based understanding of the pathomechanisms of hypertension-induced cognitive impairment.

### Electronic supplementary material

Below is the link to the electronic supplementary material.

Supplementary Material 1

## Data Availability

Anonymized and statistical information of all the participants was made available to and shared only among qualified investigators.

## Abbreviations

AD: Alzheimer’s disease
3D: three-dimensional
APOE: apolipoprotein E
Aβ: amyloid-beta
BP: blood pressure
BPV: blood pressure variability
CDK-5: cyclin-dependent kinase 5
CIs: confidence intervals
COWAT: Controlled Oral Word Association Test
CSVD: cerebral small vessel disease
CU: cognitively unimpaired
DBP: diastolic blood pressure
DM: diabetes mellitus
DSF: Digit Span Forward
FBB: 18 F-florbetaben
FLAIR: fluid-attenuated inversion recovery
FBB: 18 F-florbetaben
FMM: 18 F-flutemetamol
FTP: 18 F-flortaucipir
GSK-3β: glycogen synthase kinase 3β
HV: hippocampal volumes
ICV: intracranial volume
K-BNT: Korean version of the Boston Naming Test
MCI: mild cognitive impairment
MMSE: Mini-Mental State Examination
MRI: brain magnetic resonance imaging
NDE: natural direct effects
NIE: natural indirect effects
ORs: odds ratios
OSEM: ordered-subsets expectation-maximization
PET: positron emission tomography
PVC: partial volume correction
RBV: region-based voxel-wise correction
rdcCL: regional modified Centiloid
ROI: region of interest
RRs: risk ratios
SBP: systolic blood pressure
SD: standard deviation
SMC: Samsung Medical Center
SNSB: Seoul Neuropsychological Screening Battery
SPM: Statistical Parametric Mapping
SUVR: standardized uptake value ratio
SVLT: Seoul Verbal Learning Test
WMH: white matter hyperintensities
