# Supplementary material for: Distinct effects of blood pressure parameters on Alzheimer’s and vascular markers in 1,952 Asian individuals without dementia
Source: Alzheimers Res Ther. 2024 Jun 11;16:125. doi: 10.1186/s13195-024-01483-y (PMC11167921; doi:10.1186/s13195-024-01483-y)
Supplement: Supplementary file 1 — Supplementary Material 1 [file 13195_2024_1483_MOESM1_ESM.docx]

**Supplementary Table 1.** Multivariable linear regression analyses of Aβ uptake (in log scale) according to demographic factors and blood pressure parameters in Model 1 (without blood pressure variability) and Model 2 (with blood pressure variability)

1. Model 1

|  | **RR** | **LL** | **UL** | **p-value** |  | **RR** | **LL** | **UL** | **p-value** |
| --- | --- | --- | --- | --- | --- | --- | --- | --- | --- |
| Mean SBP  (unit: 1-SD) | 1.049 | 1.016 | 1.083 | 0.003 | Mean DBP (unit: 1-SD) | 0.973 | 0.942 | 1.004 | 0.085 |
| Age | 1.004 | 1.000 | 1.008 | 0.057 | Age | 1.004 | 1.000 | 1.008 | 0.066 |
| Education | 0.998 | 0.991 | 1.005 | 0.534 | Education | 0.997 | 0.991 | 1.004 | 0.405 |
| Gender (Male) | 1.009 | 0.947 | 1.075 | 0.780 | Gender (Male) | 1.010 | 0.947 | 1.076 | 0.768 |
| HTN | 0.960 | 0.901 | 1.023 | 0.210 | HTN | 0.992 | 0.932 | 1.057 | 0.810 |
| DM | 0.898 | 0.834 | 0.967 | 0.004 | DM | 0.888 | 0.824 | 0.958 | 0.002 |
| APOE ε4 | 1.898 | 1.784 | 2.019 | <0.0001 | APOE ε4 | 1.906 | 1.791 | 2.027 | <0.0001 |

Abbreviations: RR: risk ratio; LL: lower limit of 95% confidence interval; UL: upper limit of 95% confidence interval; SBP: systolic blood pressure; DBP: diastolic blood pressure; SD: standard deviation; HTN: hypertension; DM: diabetes mellitus; APOE ε4: apolipoprotein E ε4 allele

1. Model 2

|  | **RR** | **LL** | **UL** | **p-value** |  | **RR** | **LL** | **UL** | **p-value** |
| --- | --- | --- | --- | --- | --- | --- | --- | --- | --- |
| SD of SBP (unit: 1-SD) | 1.004 | 0.973 | 1.035 | 0.805 | SD of DBP (unit: 1-SD) | 1.011 | 0.981 | 1.042 | 0.476 |
| Mean SBP (unit: 1-SD) | 1.049 | 1.015 | 1.083 | 0.004 | Mean DBP (unit: 1-SD) | 0.972 | 0.941 | 1.003 | 0.074 |
| Age | 1.004 | 1.000 | 1.008 | 0.063 | Age | 1.004 | 1.000 | 1.008 | 0.081 |
| Education | 0.998 | 0.991 | 1.005 | 0.539 | Education | 0.997 | 0.991 | 1.004 | 0.412 |
| Gender (Male) | 1.009 | 0.947 | 1.075 | 0.784 | Gender (Male) | 1.008 | 0.946 | 1.075 | 0.798 |
| HTN | 0.959 | 0.900 | 1.023 | 0.205 | HTN | 0.991 | 0.930 | 1.055 | 0.779 |
| DM | 0.898 | 0.834 | 0.966 | 0.004 | DM | 0.888 | 0.824 | 0.957 | 0.002 |
| APOE ε4 | 1.897 | 1.784 | 2.018 | <0.0001 | APOE ε4 | 1.904 | 1.790 | 2.026 | <0.0001 |

Abbreviations: RR: risk ratio; LL: lower limit of 95% confidence interval; UL: upper limit of 95% confidence interval; SBP: systolic blood pressure; DBP: diastolic blood pressure; SD: standard deviation; HTN: hypertension; DM: diabetes mellitus; APOE ε4: apolipoprotein E ε4 allele

**Supplementary Table 2.** Multivariable logistic regression analyses of risk of WMH according to demographic factors and blood pressure parameters in Model 1 (without blood pressure variability) and Model 2 (with blood pressure variability)

1. Model

|  | **OR** | **LL** | **UL** | **p-value** |  | **OR** | **LL** | **UL** | **p-value** |
| --- | --- | --- | --- | --- | --- | --- | --- | --- | --- |
| Mean SBP (unit: 1-SD) | 1.293 | 1.015 | 1.647 | 0.038 | Mean DBP (unit: 1-SD) | 1.390 | 1.098 | 1.757 | 0.006 |
| Age | 1.038 | 1.005 | 1.073 | 0.023 | Age | 1.059 | 1.024 | 1.096 | 0.001 |
| Education | 0.959 | 0.915 | 1.006 | 0.089 | Education | 0.962 | 0.917 | 1.009 | 0.107 |
| Gender (Male) | 1.191 | 0.726 | 1.983 | 0.491 | Gender (Male) | 1.323 | 0.811 | 2.194 | 0.265 |
| HTN | 2.610 | 1.535 | 4.641 | 0.0003 | HTN | 2.654 | 1.570 | 4.691 | 0.0002 |
| DM | 1.403 | 0.850 | 2.261 | 0.181 | DM | 1.564 | 0.942 | 2.539 | 0.083 |
| APOE ε4 | 0.677 | 0.402 | 1.101 | 0.118 | APOE ε4 | 0.715 | 0.425 | 1.163 | 0.180 |

Abbreviations: OR: odds ratio; LL: lower limit of 95% confidence interval; UL: upper limit of 95% confidence interval; SBP: systolic blood pressure; DBP: diastolic blood pressure; SD: standard deviation; HTN: hypertension; DM: diabetes mellitus; APOE ε4: apolipoprotein E ε4 allele

1. Model 2

|  | **OR** | **LL** | **UL** | **p-value** |  | **OR** | **LL** | **UL** | **p-value** |
| --- | --- | --- | --- | --- | --- | --- | --- | --- | --- |
| SD of SBP (unit: 1-SD) | 1.164 | 0.931 | 1.447 | 0.181 | SD of DBP (unit: 1-SD) | 1.060 | 0.850 | 1.316 | 0.600 |
| Mean SBP (unit: 1-SD) | 1.257 | 0.984 | 1.605 | 0.067 | Mean DBP (unit: 1-SD) | 1.383 | 1.091 | 1.749 | 0.007 |
| Age | 1.035 | 1.003 | 1.070 | 0.034 | Age | 1.058 | 1.023 | 1.095 | 0.001 |
| Education | 0.960 | 0.916 | 1.007 | 0.096 | Education | 0.962 | 0.918 | 1.009 | 0.109 |
| Gender (Male) | 1.185 | 0.722 | 1.973 | 0.505 | Gender (Male) | 1.314 | 0.805 | 2.181 | 0.277 |
| HTN | 2.559 | 1.504 | 4.551 | 0.0004 | HTN | 2.638 | 1.561 | 4.663 | 0.0002 |
| DM | 1.379 | 0.835 | 2.224 | 0.204 | DM | 1.558 | 0.939 | 2.529 | 0.085 |
| APOE ε4 | 0.676 | 0.402 | 1.100 | 0.117 | APOE ε4 | 0.713 | 0.424 | 1.160 | 0.176 |

Abbreviations: OR: odds ratio; LL: lower limit of 95% confidence interval; UL: upper limit of 95% confidence interval; SBP: systolic blood pressure; DBP: diastolic blood pressure; SD: standard deviation; HTN: hypertension; DM: diabetes mellitus; APOE ε4: apolipoprotein E ε4 allele

**Supplementary Table 3.** Multivariable linear regression analyses of tau uptake according to demographic factors and blood pressure parameters in Model 1 (without blood pressure variability) and Model 2 (with blood pressure variability)

1. Model 1

|  | **Coeff** | **LL** | **UL** | **p-value** |  | **Coeff** | **LL** | **UL** | **p-value** |
| --- | --- | --- | --- | --- | --- | --- | --- | --- | --- |
| Mean SBP (unit: 1-SD) | 0.007 | -0.092 | 0.107 | 0.882 | Mean DBP (unit: 1-SD) | -0.007 | -0.101 | 0.088 | 0.888 |
| Age | -0.007 | -0.022 | 0.008 | 0.339 | Age | -0.007 | -0.021 | 0.007 | 0.334 |
| Education | 0.002 | -0.016 | 0.020 | 0.799 | Education | 0.002 | -0.016 | 0.020 | 0.812 |
| Gender (Male) | 0.106 | -0.080 | 0.292 | 0.261 | Gender (Male) | 0.108 | -0.076 | 0.293 | 0.247 |
| HTN | -0.130 | -0.324 | 0.065 | 0.189 | HTN | -0.124 | -0.320 | 0.073 | 0.215 |
| DM | 0.060 | -0.155 | 0.276 | 0.579 | DM | 0.056 | -0.165 | 0.278 | 0.616 |
| APOE ε4 | 0.116 | -0.066 | 0.298 | 0.209 | APOE ε4 | 0.116 | -0.066 | 0.299 | 0.208 |

Abbreviations: Coeff: regression coefficient; LL: lower limit of 95% confidence interval; UL: upper limit of 95% confidence interval; SBP: systolic blood pressure; DBP: diastolic blood pressure; SD: standard deviation; HTN: hypertension; DM: diabetes mellitus; APOE ε4: apolipoprotein E ε4 allele

1. Model 2

|  | **Coeff** | **LL** | **UL** | **p-value** |  | **Coeff** | **LL** | **UL** | **p-value** |
| --- | --- | --- | --- | --- | --- | --- | --- | --- | --- |
| SD of SBP (unit: 1-SD) | 0.094 | 0.001 | 0.187 | 0.049 | SD of DBP (unit: 1-SD) | 0.096 | 0.007 | 0.184 | 0.034 |
| Mean SBP (unit: 1-SD) | -0.006 | -0.105 | 0.093 | 0.911 | Mean DBP (unit: 1-SD) | -0.014 | -0.107 | 0.079 | 0.768 |
| Age | -0.009 | -0.023 | 0.006 | 0.250 | Age | -0.007 | -0.021 | 0.006 | 0.292 |
| Education | 0.006 | -0.012 | 0.024 | 0.498 | Education | 0.004 | -0.014 | 0.022 | 0.627 |
| Gender (Male) | 0.116 | -0.067 | 0.300 | 0.211 | Gender (Male) | 0.110 | -0.071 | 0.291 | 0.231 |
| HTN | -0.138 | -0.330 | 0.053 | 0.155 | HTN | -0.123 | -0.317 | 0.070 | 0.208 |
| DM | 0.036 | -0.177 | 0.250 | 0.737 | DM | 0.041 | -0.177 | 0.259 | 0.707 |
| APOE ε4 | 0.117 | -0.062 | 0.297 | 0.197 | APOE ε4 | 0.107 | -0.073 | 0.286 | 0.240 |

Abbreviations: Coeff: regression coefficient; LL: lower limit of 95% confidence interval; UL: upper limit of 95% confidence interval; SBP: systolic blood pressure; DBP: diastolic blood pressure; SD: standard deviation; HTN: hypertension; DM: diabetes mellitus; APOE ε4: apolipoprotein E ε4 allele

**Supplementary Table 4.** Multivariable linear regression analyses of hippocampal volume according to demographic factors and blood pressure parameters in Model 1 (without blood pressure variability) and Model 2 (with blood pressure variability)

A. Model 1

|  | **Coeff** | **LL** | **UL** | **p-value** |  | **Coeff** | **LL** | **UL** | **p-value** |
| --- | --- | --- | --- | --- | --- | --- | --- | --- | --- |
| Mean SBP  (unit: 1-SD) | -22.010 | -45.613 | 1.593 | 0.068 | Mean DBP  (unit: 1-SD) | 14.007 | -9.375 | 37.389 | 0.240 |
| Age | -25.202 | -28.028 | -22.377 | <0.0001 | Age | -25.162 | -28.079 | -22.246 | <0.0001 |
| Education | -0.989 | -5.993 | 4.015 | 0.698 | Education | -0.634 | -5.652 | 4.384 | 0.804 |
| Gender (Male) | -13.191 | -70.291 | 43.908 | 0.651 | Gender (Male) | -15.246 | -72.322 | 41.830 | 0.600 |
| HTN | 45.398 | -1.883 | 92.678 | 0.060 | HTN | 29.671 | -16.823 | 76.165 | 0.211 |
| DM | -33.062 | -87.766 | 21.642 | 0.236 | DM | -28.390 | -83.768 | 26.988 | 0.315 |
| ICV (m3) | 881.734 | 669.359 | 1094.109 | <0.0001 | ICV (m3) | 870.394 | 657.791 | 1082.997 | <0.0001 |

Abbreviations: Coeff: regression coefficient; LL: lower limit of 95% confidence interval; UL: upper limit of 95% confidence interval; SBP: systolic blood pressure; DBP: diastolic blood pressure; SD: standard deviation; HTN: hypertension; DM: diabetes mellitus; ICV: intracranial volume

B. Model 2

|  | **Coeff** | **LL** | **UL** | **p-value** |  | **Coeff** | **LL** | **UL** | **p-value** |
| --- | --- | --- | --- | --- | --- | --- | --- | --- | --- |
| SD of SBP (unit: 1-SD) | -21.324 | -44.156 | 1.507 | 0.067 | SD of DBP (unit: 1-SD) | -41.466 | -63.585 | -19.348 | 0.0002 |
| Mean SBP (unit: 1-SD) | -18.137 | -42.085 | 5.812 | 0.138 | Mean DBP (unit: 1-SD) | 18.225 | -5.180 | 41.630 | 0.127 |
| Age | -24.879 | -27.724 | -22.035 | <0.0001 | Age | -24.534 | -27.459 | -21.609 | <0.0001 |
| Education | -1.106 | -6.108 | 3.897 | 0.665 | Education | -0.698 | -5.698 | 4.302 | 0.784 |
| Gender (Male) | -12.887 | -69.948 | 44.175 | 0.658 | Gender (Male) | -11.101 | -68.012 | 45.811 | 0.702 |
| HTN | 47.985 | 0.655 | 95.314 | 0.047 | HTN | 34.524 | -11.873 | 80.921 | 0.145 |
| DM | -30.699 | -85.424 | 24.025 | 0.271 | DM | -27.094 | -82.275 | 28.087 | 0.336 |
| ICV (m3) | 875.596 | 663.265 | 1087.927 | <0.0001 | ICV (m3) | 864.868 | 653.018 | 1076.719 | <0.0001 |

Abbreviations: Coeff: regression coefficient; LL: lower limit of 95% confidence interval; UL: upper limit of 95% confidence interval; SBP: systolic blood pressure; DBP: diastolic blood pressure; SD: standard deviation; HTN: hypertension; DM: diabetes mellitus; ICV: intracranial volume

**Supplementary Table 5.** Multivariable linear regression analyses of MMSE according to demographic factors and blood pressure parameters in Model 1 (without blood pressure variability) and Model 2 (with blood pressure variability)

1. Model 1

|  | **Coeff** | **LL** | **UL** | **p-value** |  | **Coeff** | **LL** | **UL** | **p-value** |
| --- | --- | --- | --- | --- | --- | --- | --- | --- | --- |
| Mean SBP (unit: 1-SD) | -0.214 | -0.379 | -0.049 | 0.011 | Mean DBP (unit: 1-SD) | 0.044 | -0.120 | 0.208 | 0.597 |
| Age | -0.046 | -0.067 | -0.025 | <0.0001 | Age | -0.050 | -0.072 | -0.029 | <0.0001 |
| Education | 0.270 | 0.235 | 0.304 | <0.0001 | Education | 0.273 | 0.238 | 0.308 | <0.0001 |
| Gender (Male) | 0.016 | -0.316 | 0.349 | 0.923 | Gender (Male) | -0.001 | -0.335 | 0.334 | 0.998 |
| HTN | -0.026 | -0.359 | 0.307 | 0.877 | HTN | -0.154 | -0.482 | 0.173 | 0.355 |
| DM | -0.099 | -0.490 | 0.291 | 0.618 | DM | -0.090 | -0.485 | 0.306 | 0.657 |
| APOE ε4 | -1.083 | -1.399 | -0.768 | <0.0001 | APOE ε4 | -1.114 | -1.430 | -0.798 | <0.0001 |

Abbreviations: Coeff: regression coefficient; LL: lower limit of 95% confidence interval; UL: upper limit of 95% confidence interval; SBP: systolic blood pressure; DBP: diastolic blood pressure; SD: standard deviation; HTN: hypertension; DM: diabetes mellitus; APOE ε4: apolipoprotein E ε4 allele

1. Model 2

|  | **Coeff** | **LL** | **UL** | **p-value** |  | **Coeff** | **LL** | **UL** | **p-value** |
| --- | --- | --- | --- | --- | --- | --- | --- | --- | --- |
| SD of SBP (unit: 1-SD) | -0.050 | -0.211 | 0.111 | 0.542 | SD of DBP (unit: 1-SD) | -0.156 | -0.313 | 0.001 | 0.051 |
| Mean SBP (unit: 1-SD) | -0.205 | -0.373 | -0.036 | 0.017 | Mean DBP (unit: 1-SD) | 0.067 | -0.099 | 0.232 | 0.429 |
| Age | -0.046 | -0.067 | -0.024 | <0.0001 | Age | -0.047 | -0.069 | -0.025 | <0.0001 |
| Education | 0.269 | 0.234 | 0.304 | <0.0001 | Education | 0.273 | 0.238 | 0.307 | <0.0001 |
| Gender (Male) | 0.020 | -0.313 | 0.353 | 0.907 | Gender (Male) | 0.015 | -0.320 | 0.350 | 0.928 |
| HTN | -0.021 | -0.354 | 0.313 | 0.903 | HTN | -0.144 | -0.471 | 0.184 | 0.390 |
| DM | -0.092 | -0.484 | 0.299 | 0.643 | DM | -0.082 | -0.477 | 0.313 | 0.683 |
| APOE ε4 | -1.080 | -1.396 | -0.764 | <0.0001 | APOE ε4 | -1.096 | -1.411 | -0.780 | <0.0001 |

Abbreviations: Coeff: regression coefficient; LL: lower limit of 95% confidence interval; UL: upper limit of 95% confidence interval; SBP: systolic blood pressure; DBP: diastolic blood pressure; SD: standard deviation; HTN: hypertension; DM: diabetes mellitus; APOE ε4: apolipoprotein E ε4 allele

**Supplementary Table 6.** Multivariable linear regression analyses of Aβ uptake (in log scale) according to demographic factors and blood pressure parameters in Model 1 (without blood pressure variability) and Model 2 (with blood pressure variability) with the interaction term between blood pressure parameters and hypertension

1. Model 1

|  | **RR** | **LL** | **UL** | **p-value** |  | **RR** | **LL** | **UL** | **p-value** |
| --- | --- | --- | --- | --- | --- | --- | --- | --- | --- |
| Mean SBP  (unit: 1-SD) | 1.074 | 1.029 | 1.121 | 0.001 | Mean DBP  (unit: 1-SD) | 1.000 | 0.957 | 1.045 | 0.994 |
| Age | 1.004 | 1.000 | 1.007 | 0.066 | Age | 1.004 | 1.000 | 1.008 | 0.064 |
| Education | 0.998 | 0.992 | 1.005 | 0.593 | Education | 0.997 | 0.991 | 1.004 | 0.417 |
| Gender (Male) | 1.014 | 0.951 | 1.081 | 0.674 | Gender (Male) | 1.014 | 0.951 | 1.081 | 0.678 |
| HTN | 1.680 | 0.846 | 3.337 | 0.138 | HTN | 1.590 | 0.942 | 2.686 | 0.083 |
| DM | 0.897 | 0.833 | 0.965 | 0.004 | DM | 0.886 | 0.822 | 0.954 | 0.001 |
| APOE ε4 | 1.897 | 1.783 | 2.017 | <0.0001 | APOE ε4 | 1.904 | 1.790 | 2.025 | <0.0001 |
| **Mean SBP**  $\boldsymbol{\times}$ **HTN** | 0.950 | 0.892 | 1.011 | 0.108 | **Mean DBP**  $\boldsymbol{\times}$ **HTN** | 0.948 | 0.893 | 1.006 | 0.076 |

Abbreviations: RR: risk ratio; LL: lower limit of 95% confidence interval; UL: upper limit of 95% confidence interval; SBP: systolic blood pressure; DBP: diastolic blood pressure; SD: standard deviation; HTN: hypertension; DM: diabetes mellitus; APOE ε4: apolipoprotein E ε4 allele

1. Model 2

|  | **RR** | **LL** | **UL** | **p-value** |  | **RR** | **LL** | **UL** | **p-value** |
| --- | --- | --- | --- | --- | --- | --- | --- | --- | --- |
| SD of SBP  (unit: 1-SD) | 1.028 | 0.984 | 1.074 | 0.215 | SD of DBP  (unit: 1-SD) | 1.027 | 0.984 | 1.072 | 0.223 |
| Mean SBP  (unit: 1-SD) | 1.048 | 1.014 | 1.082 | 0.005 | Mean DBP  (unit: 1-SD) | 0.971 | 0.941 | 1.003 | 0.071 |
| Age | 1.004 | 1.000 | 1.007 | 0.072 | Age | 1.003 | 0.999 | 1.007 | 0.091 |
| Education | 0.998 | 0.992 | 1.005 | 0.591 | Education | 0.997 | 0.991 | 1.004 | 0.447 |
| Gender (Male) | 1.097 | 0.911 | 1.321 | 0.329 | Gender (Male) | 1.085 | 0.900 | 1.307 | 0.392 |
| HTN | 1.011 | 0.949 | 1.078 | 0.729 | HTN | 1.010 | 0.947 | 1.076 | 0.770 |
| DM | 0.899 | 0.835 | 0.968 | 0.005 | DM | 0.887 | 0.823 | 0.956 | 0.002 |
| APOE ε4 | 1.894 | 1.781 | 2.015 | <0.0001 | APOE ε4 | 1.901 | 1.786 | 2.022 | <0.0001 |
| **SD of SBP**  $\boldsymbol{\times}$ **HTN** | 0.955 | 0.899 | 1.014 | 0.132 | **SD of DBP**  $\boldsymbol{\times}$ **HTN** | 0.970 | 0.913 | 1.029 | 0.313 |

Abbreviations: RR: risk ratio; LL: lower limit of 95% confidence interval; UL: upper limit of 95% confidence interval; SBP: systolic blood pressure; DBP: diastolic blood pressure; SD: standard deviation; HTN: hypertension; DM: diabetes mellitus; APOE ε4: apolipoprotein E ε4 allele

**Supplementary Table 7.** Multivariable logistic regression analyses of risk of WMH according to demographic factors and blood pressure parameters in Model 1 (without blood pressure variability) and Model 2 (with blood pressure variability) with the interaction term between blood pressure parameters and hypertension

1. Model 1

|  | **OR** | **LL** | **UL** | **p-value** |  | **OR** | **LL** | **UL** | **p-value** |
| --- | --- | --- | --- | --- | --- | --- | --- | --- | --- |
| Mean SBP  (unit: 1-SD) | 0.988 | 0.614 | 1.584 | 0.958 | Mean DBP  (unit: 1-SD) | 1.294 | 0.798 | 2.080 | 0.293 |
| Age | 1.039 | 1.006 | 1.074 | 0.019 | Age | 1.059 | 1.024 | 1.096 | 0.001 |
| Education | 0.957 | 0.913 | 1.004 | 0.074 | Education | 0.962 | 0.917 | 1.009 | 0.107 |
| Gender (Male) | 1.162 | 0.707 | 1.936 | 0.557 | Gender (Male) | 1.316 | 0.806 | 2.183 | 0.275 |
| HTN | 0.050 | 0.000 | 19.926 | 0.324 | HTN | 1.156 | 0.011 | 138.353 | 0.952 |
| DM | 1.417 | 0.858 | 2.287 | 0.170 | DM | 1.569 | 0.945 | 2.547 | 0.081 |
| APOE ε4 | 0.683 | 0.406 | 1.112 | 0.128 | APOE ε4 | 0.716 | 0.425 | 1.166 | 0.183 |
| **Mean SBP**  $\boldsymbol{\times}$ **HTN** | 1.434 | 0.833 | 2.476 | 0.194 | **Mean DBP**  $\boldsymbol{\times}$ **HTN** | 1.097 | 0.644 | 1.879 | 0.735 |

Abbreviations: OR: odds ratio; LL: lower limit of 95% confidence interval; UL: upper limit of 95% confidence interval; SBP: systolic blood pressure; DBP: diastolic blood pressure; SD: standard deviation; HTN: hypertension; DM: diabetes mellitus; APOE ε4: apolipoprotein E ε4 allele

1. Model 2

|  | **OR** | **LL** | **UL** | **p-value** |  | **OR** | **LL** | **UL** | **p-value** |
| --- | --- | --- | --- | --- | --- | --- | --- | --- | --- |
| SD of SBP  (unit: 1-SD) | 1.653 | 1.097 | 2.416 | 0.018 | SD of DBP  (unit: 1-SD) | 1.200 | 0.756 | 1.794 | 0.423 |
| Mean SBP  (unit: 1-SD) | 1.251 | 0.979 | 1.597 | 0.073 | Mean DB  P (unit: 1-SD) | 1.380 | 1.089 | 1.746 | 0.008 |
| Age | 1.034 | 1.001 | 1.069 | 0.041 | Age | 1.057 | 1.022 | 1.094 | 0.001 |
| Education | 0.963 | 0.918 | 1.010 | 0.120 | Education | 0.963 | 0.918 | 1.010 | 0.117 |
| Gender (Male) | 1.201 | 0.732 | 1.999 | 0.003 | Gender (Male) | 1.317 | 0.807 | 2.186 | 0.087 |
| HTN | 11.350 | 2.269 | 60.022 | 0.472 | HTN | 4.194 | 0.815 | 21.609 | 0.273 |
| DM | 1.383 | 0.838 | 2.230 | 0.200 | DM | 1.553 | 0.936 | 2.519 | 0.087 |
| APOE ε4 | 0.667 | 0.396 | 1.086 | 0.105 | APOE ε4 | 0.707 | 0.420 | 1.152 | 0.167 |
| **SD of SBP**  $\boldsymbol{\times}$ **HTN** | 0.624 | 0.396 | 1.003 | 0.051 | **SD of DBP**  $\boldsymbol{\times}$ **HTN** | 0.855 | 0.532 | 1.438 | 0.544 |

Abbreviations: OR: odds ratio; LL: lower limit of 95% confidence interval; UL: upper limit of 95% confidence interval; SBP: systolic blood pressure; DBP: diastolic blood pressure; SD: standard deviation; HTN: hypertension; DM: diabetes mellitus; APOE ε4: apolipoprotein E ε4 allele

**Supplementary Table 8.** Multivariable linear regression analyses of tau uptake according to demographic factors and blood pressure parameters in Model 1 (without blood pressure variability) and Model 2 (with blood pressure variability) with the interaction term between blood pressure parameters and hypertension

1. Model 1

|  | **Coeff** | **LL** | **UL** | **p-value** |  | **Coeff** | **LL** | **UL** | **p-value** |
| --- | --- | --- | --- | --- | --- | --- | --- | --- | --- |
| Mean SBP  (unit: 1-SD) | -0.001 | -0.145 | 0.143 | 0.989 | Mean DBP  (unit: 1-SD) | -0.002 | -0.157 | 0.153 | 0.978 |
| Age | -0.007 | -0.022 | 0.008 | 0.345 | Age | -0.007 | -0.021 | 0.007 | 0.338 |
| Education | 0.002 | -0.016 | 0.020 | 0.799 | Education | 0.002 | -0.016 | 0.021 | 0.807 |
| Gender (Male) | 0.107 | -0.080 | 0.294 | 0.260 | Gender (Male) | 0.110 | -0.080 | 0.300 | 0.255 |
| HTN | -0.300 | -2.393 | 1.794 | 0.777 | HTN | -0.045 | -2.188 | 2.098 | 0.967 |
| DM | 0.064 | -0.157 | 0.286 | 0.566 | DM | 0.054 | -0.173 | 0.282 | 0.636 |
| APOE ε4 | 0.117 | -0.067 | 0.300 | 0.210 | APOE ε4 | 0.116 | -0.069 | 0.301 | 0.218 |
| **Mean SBP**  $\boldsymbol{\times}$ **HTN** | 0.015 | -0.174 | 0.205 | 0.872 | **Mean DBP**  $\boldsymbol{\times}$ **HTN** | -0.007 | -0.210 | 0.195 | 0.942 |

Abbreviations: Coeff: regression coefficient; LL: lower limit of 95% confidence interval; UL: upper limit of 95% confidence interval; SBP: systolic blood pressure; DBP: diastolic blood pressure; SD: standard deviation; HTN: hypertension; DM: diabetes mellitus; APOE ε4: apolipoprotein E ε4 allele

1. Model 2

|  | **Coeff** | **LL** | **UL** | **p-value** |  | **Coeff** | **LL** | **UL** | **p-value** |
| --- | --- | --- | --- | --- | --- | --- | --- | --- | --- |
| SD of SBP  (unit: 1-SD) | 0.145 | 0.016 | 0.274 | 0.029 | SD of DBP  (unit: 1-SD) | 0.132 | 0.001 | 0.263 | 0.049 |
| Mean SBP  (unit: 1-SD) | -0.005 | -0.104 | 0.094 | 0.925 | Mean DBP  (unit: 1-SD) | -0.016 | -0.110 | 0.077 | 0.728 |
| Age | -0.009 | -0.024 | 0.006 | 0.230 | Age | -0.007 | -0.021 | 0.006 | 0.297 |
| Education | 0.008 | -0.010 | 0.027 | 0.372 | Education | 0.005 | -0.013 | 0.023 | 0.583 |
| Gender (Male) | 0.130 | -0.054 | 0.315 | 0.499 | Gender (Male) | 0.118 | -0.065 | 0.300 | 0.793 |
| HTN | 0.232 | -0.447 | 0.911 | 0.165 | HTN | 0.075 | -0.492 | 0.642 | 0.204 |
| DM | 0.059 | -0.158 | 0.277 | 0.588 | DM | 0.056 | -0.166 | 0.278 | 0.618 |
| APOE ε4 | 0.110 | -0.069 | 0.290 | 0.225 | APOE ε4 | 0.117 | -0.065 | 0.299 | 0.205 |
| **SD of SBP**  $\boldsymbol{\times}$ **HTN** | -0.105 | -0.290 | 0.080 | 0.261 | **SD of DBP**  $\boldsymbol{\times}$ **HTN** | -0.068 | -0.251 | 0.115 | 0.461 |

Abbreviations: Coeff: regression coefficient; LL: lower limit of 95% confidence interval; UL: upper limit of 95% confidence interval; SBP: systolic blood pressure; DBP: diastolic blood pressure; SD: standard deviation; HTN: hypertension; DM: diabetes mellitus; APOE ε4: apolipoprotein E ε4 allele

**Supplementary Table 9.** Multivariable linear regression analyses of hippocampal volume according to demographic factors and blood pressure parameters in Model 1 (without blood pressure variability) and Model 2 (with blood pressure variability) with the interaction term between blood pressure parameters and hypertension

1. Model 1

|  | **Coeff** | **LL** | **UL** | **p-value** |  | **Coeff** | **LL** | **UL** | **p-value** |
| --- | --- | --- | --- | --- | --- | --- | --- | --- | --- |
| Mean SBP  (unit: 1-SD) | -35.326 | -66.674 | -3.978 | 0.027 | Mean DBP  (unit: 1-SD) | 4.790 | -27.707 | 37.286 | 0.773 |
| Age | -25.130 | -27.957 | -22.303 | <0.0001 | Age | -25.168 | -28.084 | -22.251 | <0.0001 |
| Education | -1.180 | -6.192 | 3.832 | 0.644 | Education | -0.650 | -5.669 | 4.368 | 0.799 |
| Gender (Male) | -15.536 | -72.741 | 41.668 | 0.594 | Gender (Male) | -16.524 | -73.688 | 40.641 | 0.571 |
| HTN | -282.094 | -790.841 | 226.654 | 0.277 | HTN | -130.492 | -519.121 | 258.136 | 0.510 |
| DM | -32.536 | -87.236 | 22.164 | 0.244 | DM | -27.212 | -82.668 | 28.244 | 0.336 |
| ICV (m3) | 884.126 | 671.756 | 1096.495 | <0.0001 | ICV (m3) | 870.172 | 657.547 | 1082.797 | <0.0001 |
| **Mean SBP**  $\boldsymbol{\times}$ **HTN** | 29.989 | -16.397 | 76.375 | 0.205 | **Mean DBP**  $\boldsymbol{\times}$ **HTN** | 18.309 | -25.797 | 62.415 | 0.416 |

Abbreviations: Coeff: regression coefficient; LL: lower limit of 95% confidence interval; UL: upper limit of 95% confidence interval; SBP: systolic blood pressure; DBP: diastolic blood pressure; SD: standard deviation; HTN: hypertension; DM: diabetes mellitus; ICV: intracranial volume

1. Model 2

|  | **Coeff** | **LL** | **UL** | **p-value** |  | **Coeff** | **LL** | **UL** | **p-value** |
| --- | --- | --- | --- | --- | --- | --- | --- | --- | --- |
| SD of SBP  (unit: 1-SD) | -17.313 | -49.620 | 14.994 | 0.293 | SD of DBP  (unit: 1-SD) | -51.862 | -82.950 | -20.774 | 0.001 |
| Mean SBP  (unit: 1-SD) | -18.155 | -41.990 | 5.681 | 0.135 | Mean DBP  (unit: 1-SD) | 18.519 | -5.035 | 42.073 | 0.123 |
| Age | -24.899 | -27.747 | -22.052 | <0.0001 | Age | -24.464 | -27.393 | -21.535 | <0.0001 |
| Education | -1.060 | -6.070 | 3.950 | 0.678 | Education | -0.858 | -5.869 | 4.153 | 0.737 |
| Gender (Male) | -12.383 | -69.528 | 44.761 | 0.310 | Gender (Male) | -12.170 | -69.125 | 44.785 | 0.686 |
| HTN | 71.226 | -66.339 | 208.791 | 0.671 | HTN | -28.196 | -165.126 | 108.735 | 0.675 |
| DM | -30.442 | -85.199 | 24.316 | 0.276 | DM | -26.562 | -81.755 | 28.631 | 0.345 |
| ICV (m3) | 876.156 | 663.748 | 1088.564 | <0.0001 | ICV (m3) | 863.585 | 651.713 | 1075.457 | <0.0001 |
| **SD of SBP**  $\boldsymbol{\times}$ **HTN** | -8.023 | -52.613 | 36.566 | 0.724 | **SD of DBP**  $\boldsymbol{\times}$ **HTN** | 21.239 | -22.388 | 64.867 | 0.340 |

Abbreviations: Coeff: regression coefficient; LL: lower limit of 95% confidence interval; UL: upper limit of 95% confidence interval; SBP: systolic blood pressure; DBP: diastolic blood pressure; SD: standard deviation; HTN: hypertension; DM: diabetes mellitus; ICV: intracranial volume

**Supplementary Table 10.** Multivariable linear regression analyses of MMSE according to demographic factors and blood pressure parameters in Model 1 (without blood pressure variability) and Model 2 (with blood pressure variability) with the interaction term between blood pressure parameters and hypertension

1. Model 1

|  | **Coeff** | **LL** | **UL** | **p-value** |  | **Coeff** | **LL** | **UL** | **p-value** |
| --- | --- | --- | --- | --- | --- | --- | --- | --- | --- |
| Mean SBP  (unit: 1-SD) | -0.148 | -0.365 | 0.069 | 0.181 | Mean DBP  (unit: 1-SD) | -0.053 | -0.280 | 0.174 | 0.648 |
| Age | -0.047 | -0.068 | -0.026 | <0.0001 | Age | -0.050 | -0.072 | -0.029 | <0.0001 |
| Education | 0.270 | 0.236 | 0.305 | <0.0001 | Education | 0.272 | 0.237 | 0.307 | <0.0001 |
| Gender (Male) | 0.034 | -0.301 | 0.368 | 0.844 | Gender (Male) | -0.018 | -0.354 | 0.318 | 0.915 |
| HTN | 1.686 | -1.973 | 5.344 | 0.366 | HTN | -1.836 | -4.576 | 0.905 | 0.189 |
| DM | -0.101 | -0.491 | 0.290 | 0.613 | DM | -0.072 | -0.469 | 0.324 | 0.722 |
| APOE ε4 | -1.087 | -1.403 | -0.771 | <0.0001 | APOE ε4 | -1.112 | -1.428 | -0.796 | <0.0001 |
| **Mean SBP**  $\boldsymbol{\times}$ **HTN** | -0.154 | -0.480 | 0.173 | 0.357 | **Mean DBP**  $\boldsymbol{\times}$ **HTN** | 0.191 | -0.118 | 0.500 | 0.226 |

Abbreviations: Coeff: regression coefficient; LL: lower limit of 95% confidence interval; UL: upper limit of 95% confidence interval; SBP: systolic blood pressure; DBP: diastolic blood pressure; SD: standard deviation; HTN: hypertension; DM: diabetes mellitus; APOE ε4: apolipoprotein E ε4 allele

1. Model 2

|  | **Coeff** | **LL** | **UL** | **p-value** |  | **Coeff** | **LL** | **UL** | **p-value** |
| --- | --- | --- | --- | --- | --- | --- | --- | --- | --- |
| SD of SBP  (unit: 1-SD) | 0.018 | -0.203 | 0.240 | 0.871 | SD of DBP  (unit: 1-SD) | -0.174 | -0.391 | 0.043 | 0.116 |
| Mean SBP  (unit: 1-SD) | -0.206 | -0.375 | -0.038 | 0.016 | Mean DBP  (unit: 1-SD) | 0.067 | -0.098 | 0.233 | 0.424 |
| Age | -0.046 | -0.067 | -0.025 | <0.0001 | Age | -0.047 | -0.069 | -0.025 | <0.0001 |
| Education | 0.269 | 0.235 | 0.304 | <0.0001 | Education | 0.273 | 0.238 | 0.307 | <0.0001 |
| Gender (Male) | 0.024 | -0.309 | 0.357 | 0.887 | Gender (Male) | 0.015 | -0.320 | 0.350 | 0.929 |
| HTN | 0.406 | -0.604 | 1.416 | 0.430 | HTN | -0.264 | -1.318 | 0.790 | 0.624 |
| DM | -0.084 | -0.476 | 0.307 | 0.673 | DM | -0.081 | -0.476 | 0.315 | 0.689 |
| APOE ε4 | -1.082 | -1.398 | -0.766 | <0.0001 | APOE ε4 | -1.095 | -1.411 | -0.779 | <0.0001 |
| **SD of SBP**  $\boldsymbol{\times}$ **HTN** | -0.139 | -0.451 | 0.172 | 0.380 | **SD of DBP**  $\boldsymbol{\times}$ **HTN** | 0.037 | -0.272 | 0.347 | 0.814 |

Abbreviations: Coeff: regression coefficient; LL: lower limit of 95% confidence interval; UL: upper limit of 95% confidence interval; SBP: systolic blood pressure; DBP: diastolic blood pressure; SD: standard deviation; HTN: hypertension; DM: diabetes mellitus; APOE ε4: apolipoprotein E ε4 allele
